# Correlation of genetic alterations by whole-exome sequencing with clinical outcomes of glioblastoma patients from the Lebanese population

**DOI:** 10.1371/journal.pone.0242793

**Published:** 2020-11-25

**Authors:** Fadi S. Saadeh, Rami Z. Morsi, Abdallah El-Kurdi, Georges Nemer, Rami Mahfouz, Maya Charafeddine, Jessica Khoury, Marwan W. Najjar, Pierre Khoueiry, Hazem I. Assi

**Affiliations:** 1 Department of Neurology, Memorial Sloan Kettering Cancer Center, New York, New York, United States of America; 2 Department of Neurology, University of Chicago, Chicago, Illinois, United States of America; 3 Department of Biochemistry and Molecular Genetics, American University of Beirut, Beirut, Lebanon; 4 Department of Pathology and Laboratory Medicine, American University of Beirut Medical Center, Beirut, Lebanon; 5 Division of Hematology and Oncology, Department of Internal Medicine, American University of Beirut Medical Center, Beirut, Lebanon; 6 Division of Neurosurgery, Department of Surgery, American University of Beirut Medical Center, Beirut, Lebanon; Instituto de Investigacion Sanitaria INCLIVA, SPAIN

## Abstract

**Introduction:**

Glioblastoma (GBM) is an aggressive brain tumor associated with high degree of resistance to treatment. Given its heterogeneity, it is important to understand the molecular landscape of this tumor for the development of more effective therapies. Because of the different genetic profiles of patients with GBM, we sought to identify genetic variants in Lebanese patients with GBM (LEB-GBM) and compare our findings to those in the Cancer Genome Atlas (TCGA).

**Methods:**

We performed whole exome sequencing (WES) to identify somatic variants in a cohort of 60 patient-derived GBM samples. We focused our analysis on 50 commonly mutated GBM candidate genes and compared mutation signatures between our population and publicly available GBM data from TCGA. We also cross-tabulated biological covariates to assess for associations with overall survival, time to recurrence and follow-up duration.

**Results:**

We included 60 patient-derived GBM samples from 37 males and 23 females, with age ranging from 3 to 80 years (mean and median age at diagnosis were 51 and 56, respectively). Recurrent tumor formation was present in 94.8% of patients (n = 55/58). After filtering, we identified 360 somatic variants from 60 GBM patient samples. After filtering, we identified 360 somatic variants from 60 GBM patient samples. Most frequently mutated genes in our samples included *ATRX*, *PCDHX11*, *PTEN*, *TP53*, *NF1*, *EGFR*, *PIK3CA*, and *SCN9A*. Mutations in *NLRP5* were associated with decreased overall survival among the Lebanese GBM cohort (p = 0.002). Mutations in *NLRP5* were associated with decreased overall survival among the Lebanese GBM cohort (p = 0.002). *EGFR* and *NF1* mutations were associated with the frontal lobe and temporal lobe in our LEB-GBM cohort, respectively.

**Conclusions:**

Our WES analysis confirmed the similarity in mutation signature of the LEB-GBM population with TCGA cohorts. It showed that 1 out of the 50 commonly GBM candidate gene mutations is associated with decreased overall survival among the Lebanese cohort. This study also highlights the need for studies with larger sample sizes to inform clinicians for better prognostication and management of Lebanese patients with GBM.

## Introduction

Glioblastoma (GBM) is a malignant brain tumor, most commonly known for its aggressive nature, resistance to treatment and a median survival of approximately 1 year [1, 2]. GBM, classified as a World Health Organization (WHO) Grade IV astrocytoma, is considered the most common lethal intracranial malignancy in adults. The majority of primary GBM tumors arise de novo in the absence of a previously present lower grade lesion and are usually clinically evident within 3 months [3]. On the other hand, secondary GBMs develop from WHO Grade II (low-grade) or WHO Grade III (anaplastic astrocytoma) lesions and present with a slower progression rate than their primary counterparts. Secondary GBMs are usually more prevalent in the younger age groups and associated with a better prognosis [3–5].

The presentation and prognosis of GBM varies greatly among patients. The differences among individuals with GBM suggest that clinical and histologic characteristics greatly influence tumor invasiveness, treatment response and survival [6]. It is therefore imperative for physicians and researchers to understand the pathophysiology of GBM progression and treatment resistance in order to improve clinical outcomes [7, 8].

There have been recent advances in the understanding of the molecular pathogenesis behind GBM with the rise of next-generation sequencing methods [9–12]. Better understanding of the molecular basis of GBM has led to the development of several diagnostic, predictive and prognostic biomarkers. Some of these include O6-methylguanine-DNA methyltransferase (MGMT), epidermal growth factor receptor (EGFR), vascular endothelial growth factor (VEGF), tumor protein p53 (TP53), isocitrate dehydrogenase (IDH), phosphatase and tensin homolog (PTEN), loss of heterozygosity (LOH) 10q, LOH 10q25-qter and p16^INK4a^. Because there are differences in the genetic profiles among GBM patients, these molecular signatures have tailored treatment options and have contributed to the discovery of novel anti-GBM therapies, including but not limited to, small-molecule inhibitors, antibody-based drug conjugates, vaccines, and more recently, immune checkpoint inhibitors [13].

Because the clinical and molecular characteristics vary among individuals and populations with GBM, we sought to identify driver mutations and molecular targets using the Illumina HiSeq next-generation sequencing platform in the Lebanese GBM population (LEB-GBM). In this study, we identified 360 somatic variants from 60 patients with GBM and compared our whole exome sequencing (WES) data for 50 commonly mutated GBM candidate genes to that obtained from data generated by the Cancer Genome Atlas (TCGA) Research Network.

## Materials and methods

### Data collection

We accessed medical records for 60 GBM patients from June 2015 to June 2016, and retrospectively collected clinical data on these patients with a diagnosis date ranging from May 2003 to August 2014. This study was approved by the Institutional Review Board (IRB) of the American University of Beirut, and informed oral consent was obtained from all patients prior to sample processing via telephone call and documented on the data collection sheet. Written consent was waived by IRB because there was minimal risk to the subjects, and the study mainly involved chart review and data collection with no identifiers.

### Tumor samples

Based on the histological diagnosis performed in the Department of Pathology and Laboratory Medicine at the American University of Beirut Medical Center, 60 paraffin-embedded tissues were retrieved, and the blocks were submitted to the Molecular Diagnostics Laboratory for processing and DNA extraction.

### DNA extraction

Eight 10 μm ribbons were obtained from each block and placed in an autoclaved 1.5 mL microfuge tube. Qiagen kit was used for DNA extraction. Briefly, cell lysis was followed by incubation at 65°C and DNA precipitation by centrifugation at 13000g for 10 minutes, at 4°C. After the supernatant was discarded, DNA was eluted in 30 μL of elution buffer (10 mM Tris buffer, pH 8.5; Qiagen). The resultant DNA was then quantified using the Biomate TM spectrophotometer and stored at 4°C.

### Next generation sequencing and data processing

Extracted DNA was shipped for paired-end sequencing (Macrogen Inc., Seoul, Korea) using SureSelect Human All Exon V6 r2 as capture method on Illumina HiSeq platform with 101 bp read length at an average depth of 41x (median = 30x). Quality check on raw sequencing reads was performed using FastQC followed by reads trimming and mapping to the human genome hg38 assembly using the Burrows-Wheeler Aligner (BWA) [14]. All 60 samples passed quality check control with an average quality score of 39.26 (Min = 31.38). Aligned reads were marked for duplication followed by local realignment using the Genome Analysis Toolkit (GATK4) [15]. Somatic variant calling was performed on recalibrated reads from the previous step as input on each sample independently using Mutect2 [16] with the following parameters (Mutect2 -R hg38.fa -I sample -tumor sample.bam -O sample.vcf).

Annotation was performed using the Variant Effect Predictor (VEP, v94) and variants were classified into one of the following eight different classes ("Frame_Shift_Del", "Missense_Mutation", "Nonsense_Mutation", "Multi_Hit", "Frame_Shift_Ins", "In_Frame_Ins", "Splice_Site", "In_Frame_Del") [17]. Additionally, and to overcome false positive calls due to the lack of a reference genome for the Lebanese population, we focused our analysis on a set of 50 GBM related genes compiled from the literature ("*OR5D18*", "*UGT2B4*", "*SPTA1*", "*ZNF99*", "*OR5P2*", "*PIK3C2G*", "*ADAM29*", "*LRRC55*", "*OR5D13*", "*OR5W2*", "*EGFR*", "*IL4R*", "*SLC26A3*", "*IDH1*", "*PSG8*", "*PTEN*", "*NF1*", "*NLRP5*", "*TP53*", "*CDKN2A*", "*PDGFRA*", "*COL1A2*", "*KEL*", "*PIK3R1*", "*SEMA3C*", "*ABCB1*", "*CALCR*", "*LZTR1*", "*OR5AR1*", "*PCDH11X*", "*SCN9A*", "*SEMA3E*", "*SEMG1*", "*UGT2A3*", "*ANO2*", "*BRAF*", "*CDH18*", "*GABRA6*", "*IL18RAP*", "*LRFN5*", "*LUM*", "*OR8K3*", "*PIK3CA*", "*RPL5*", "*STAG2*", "*TERT*", "*H3F3A*", "*HIST1H3B*", "*ATRX*", "*IDH2*").

Furthermore, variants with a FILTER classification as “common_variant” were excluded. This led to a final set of 360 variants from 46 of the 50 genes mentioned above with an average depth of 92x (median = 57x) (S3 Table).

The oncoplot and its associated analysis were performed using the maftools package [18]. Clinical annotations including gender, multifocality, age, resection status, time to death and time to recurrence were integrated into the oncoplot for visualization.

### Statistical analysis

Statistical analysis was performed to analyze the quantitative data using the Statistical Package for Social Sciences (SPSS) v.26. The clinical and biological covariates were cross-tabulated and Chi-square tests and/or Fisher’s tests were used when necessary to check for statistically significant differences. The statistical tests were two-sided, with a *p*-value of < 0.05 considered statistically significant. Student t-test was used to compare the difference in age, tumor size, mean duration of overall survival, time to tumor recurrence and follow-up duration between samples with specific gene mutations and those without these specific gene mutations. Data for the commonly mutated GBM genes in the LEB-GBM cohort was then compared to GBM “Mutations” data from TCGA that can be found in “Explore Project Data” under the link https://portal.gdc.cancer.gov/projects/TCGA-GBM.

## Results

### Patient demographics and clinical characteristics

A cohort of 60 samples from 37 males and 23 females was included in study, with age ranging from 3 to 80 years (mean and median age at diagnosis were 51 and 56, respectively) (S1 Table). The male-to-female ratio was 1.6 to 1 with a mean overall survival of 17.3 months (n = 50/60). The majority of the tumors were found in the cerebral hemispheres, with the frontal region being the most common location (40.0%; n = 24/60). Retrospectively, based on the profile of *IDH1* mutations, most tumors were primary in origin (85.0%; n = 51/60), while the remaining tumors were secondary in origin (15.0%; n = 9/60). Approximately 35.6% of patients underwent resection (n = 21/59), and around 92.0% of patients received adjuvant temozolomide (TMZ) treatment (n = 46/50). All patients received radiotherapy (100.0%; n = 60/60) with the majority receiving concurrent TMZ (88.1%; n = 52/59). Recurrent tumor formation was present in 94.8% of patients (n = 55/58). The average time to recurrence was 10.6 months (n = 47/60). The mean follow-up duration for patients was 19.8 months (n = 60/60).

### Distribution of frequently mutated genes across LEB-GBM patients

**Fig 1:**
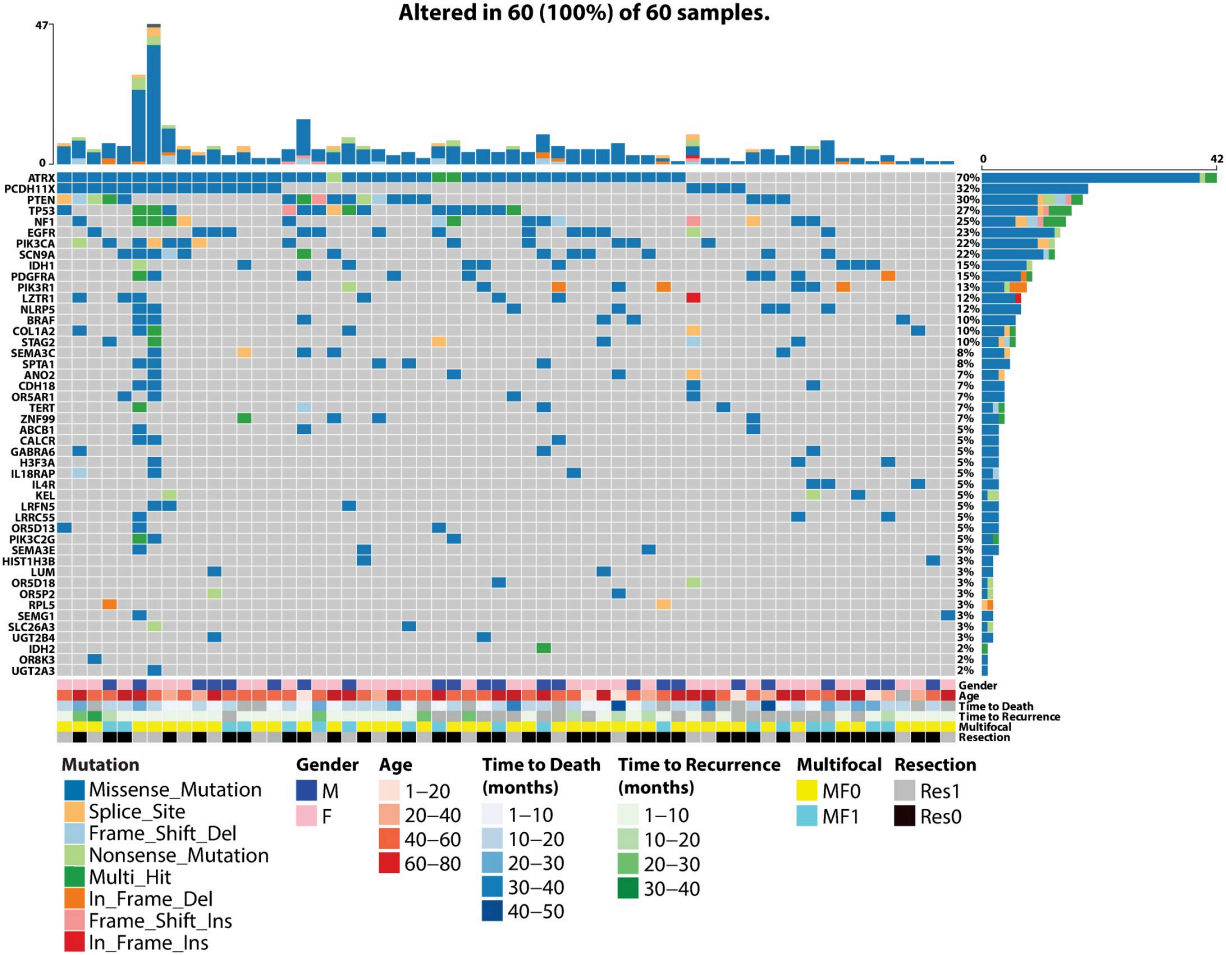
Mutational landscape of LEB-GBM cohort. Oncoplot of the 46 remaining candidate genes (rows), after filtering, commonly mutated in GBM. Columns correspond to samples. Colored squares indicate mutated genes and grey squares indicate non-mutated genes. Each color represents a different type of mutation: missense mutation (blue), splice site mutation (light orange), frame shift deletion (light blue), nonsense mutation (light green), multi-hit (dark green), in frame deletion (dark orange), frame shift insertion (pink) and in frame insertion (red). Percentages represent mutation rate among all LEB-GBM patient samples.

Allele frequencies vary between populations and are considered a major source of phenotypic divergence. In order to characterize the mutational signature of GBM in the Lebanese population, we performed Whole Exome Sequencing (WES) on the cohort of 60 GBM patients described above. Variant calling from our WES analysis identified a total of 561,540 mutations and small insertions and deletions (INDELs) (Median = 9,543 alterations per sample) with a majority of 92.6% missense mutations (S2 Table). With the lack of a reference genome for the Lebanese population, the majority of the identified mutations may correspond to false positive calls. For this, we decided to limit our analysis to all non-common variants in a list of 50 frequently mutated genes in GBM (Materials and Methods). Our selection and filtering approaches reduced the number of called mutations to 360 non-common mutations affecting 46 out of the 50 genes in our list, including 292 (81.1%) missense mutations in 60 samples (Fig 1, S3 Table). Four genes (“*ADAM29*”, “*OR5W2*”, “*PSG8*” and “*CDKN2A*”) did not show any mutation in any of the samples following our filtering approach for non-common variants.

Genes most frequently mutated in our samples include those with extensively studied and established roles in GBM and frequencies greater than or equal to 10.0% like *ATRX* (70.0%, 95% CI: 58.4–81.6; n = 42/60), followed by *PCDHX11* (31.7%, 95% CI: 19.9–43.4; n = 19/60), *PTEN* (30.0%, 95% CI: 18.4–41.6; n = 18/60), *TP53* (26.7%, 95% CI: 15.5–37.9; n = 16/60), *NF1* (25.0%, 95% CI: 14.0–36.0; n = 15/60), *EGFR* (23.3%, 95% CI: 12.6–34.0; n = 14/60), *PIK3CA* (21.7%, 95% CI: 11.2–32.1; n = 13/60), *LZTR1* (11.7%, 95% CI: 3.5–19.8; n = 7/60), *BRAF* (10.0%, 95% CI: 2.4–17.6; n = 6/60), and *COL1A2* (10.0%, 95% CI: 2.4–17.6; n = 6/60) (Fig 1). Other genes less extensively studied in the context of GBM but also had frequencies greater than or equal to 10.0% include *SCN9A* (21.7%, 95% CI: 11.2–32.1; n = 13/60), *NLRP5* (11.7%, 95% CI: 3.5–19.8; n = 7/60), and *STAG2* (10.0%, 95% CI: 2.4–17.6; n = 6/60). Despite a frequency of less than 10.0%, it is noteworthy to mention that mutations in the *TERT* gene accounted for 6.7% of our samples (95% CI: 0.4–13.0; n = 4/60). As the capture method is mostly exclusive to coding regions (Materials and Methods), known GBM associated mutations in the TERT promoter (C228T or C250T) were not assayed.

The most frequently mutated gene in our LEB-GBM cohort is *ATRX* (alpha-thalassemia/mental retardation, X-linked) with 70.0% (95% CI: 58.4–81.6; n = 42/60) samples affected by 16 different types of missense variants. More specifically, the most frequent mutation in *ATRX* is an E929Q mutation (rs3088074; 63.3%, 95% CI: 51.1–75.5; n = 38/60) (S3 and S4 Tables) with a benign clinical significance and characterized with a population frequency varying from 33.1% (n = 3695/11176, ALFA Project) to 49.3% (n = 61923/125568, TOPMED) [19].

Other observations included were R175H, R248Q and R337C mutations being the most common missense *TP53* mutations in the LEB-GBM cohort, each comprising 3.3% (95% CI: -1.2–7.9; n = 2/60) of the samples (Table 1, S3 and S4 Tables). The most common *EGFR* mutations in our study were G598V (5.0%, 95% CI: -0.5–10.5; n = 3/60), A289T (3.3%, 95% CI: -1.2–7.9; n = 2/60) and R108K (3.3%, 95% CI: -1.2–7.9; n = 2/60) missense mutations (Table 1, S3 and S4 Tables). There was no significant association between concurrent *TP53* and *EGFR* mutations (21.4%; n = 3/14; p = 0.537). The most common *PIK3CA* mutation was the H1047R missense mutation (3.3%, 95% CI: -1.2–7.9; n = 2/60). The most common *SCN9A* mutations were E1974K (6.7%, 95% CI: 0.4–13.0; n = 4/60), S1972R (5.0%, 95% CI: -0.5–10.5; n = 3/60) and S1975K (3.3%, 95% CI: -1.2–7.9; n = 2/60) missense mutations (S3 and S4 Tables). There was no significant association between concurrent *SCN9A* and *IDH1* mutations (p = 0.999). There was also no significant association between concurrent *IDH1* and *TP53* mutations (p = 0.065).

**Table 1:** Comparison of common GBM gene mutations between the LEB-GBM cohort and the TCGA cohort.

Gene	Mutation	Type of Mutation	# Affected Samples (n = 60)	% Affected Samples (n = 60)^a^	95% CI (LEB-GBM Cohort)^a^	# TCGA (n = 391)	% TCGA (n = 391)^b^	95% CI (TCGA Cohort)^b^
***IDH1***	*R132H*	Missense	6	10.0	2.4–17.6	23	5.9	3.6–8.2
**EGFR**	*A289V*	Missense	1	1.7	-1.6–4.9	15	3.8	1.9–5.7
***EGFR***	*G598V*	Missense	3	5.0	-0.5–10.5	13	3.3	1.5–5.1
***TP53***	*R175H*	Missense	2	3.3	-1.2–7.9	8	2.0	0.6–3.4
***TP53***	*R248Q*	Missense	2	3.3	1.1–5.8	7	1.8	0.5–3.1
**EGFR**	*R222C*	Missense	0	0.0	0.0–0.0	6	1.5	0.3–2.8
**TP53**	R248W	Missense	1	1.7	-1.6–4.9	5	1.3	0.2–2.4
**TP53**	R282W	Missense	0	0.0	0.0–0.0	5	1.3	0.2–2.4
**EGFR**	*A289D*	Missense	0	0.0	0.0–0.0	5	1.3	0.2–2.4
**BRAF**	*V600E*	Missense	2	3.3	-1.2–7.9	5	1.3	0.2–2.4

^a^ The denominator used for the percentages values in these columns was 60, reflective of the number of samples tested

^b^ The denominator used for the percentages values in these columns was 391, reflective of the number of cases tested for gene mutations

### Distribution of frequent mutations in the LEB-GBM cohort

Following assessment of frequently mutated genes in our GBM samples, we interrogated recurrent mutations in our cohort and cross-compared their frequencies between our population and cohorts obtained from TCGA.

The most prevalent mutation in GBM, *IDH1* R132H, showed a higher mutation frequency in our samples (10.0%, 95% CI: 2.4–17.6) compared to its frequency in TCGA (5.9%, 95% CI: 3.6–8.2) (Table 1, S3 and S4 Tables). Although it can be due to the lower sample size in our cohort, it may reflect a more prevalent role of *IDH1* R132H mutation in the Lebanese population. Recurrent mutations in *EGFR*, *TP53* and *BRAF* showed similar frequencies between our cohort and TCGA with 5.0%, 3.3%, 3.3%, 3.3%, and 1.7% for *EGFR* G598V, *TP53* R175H, *TP53* R248Q, *BRAF* V600E, and *TP53* R248W mutations respectively (Table 1). However, *EGFR* A289V mutation had a frequency of 1.7% (95% CI: -1.6–4.9; n = 1/60), lower than its frequency in TCGA (3.8%, 95% CI: 1.9–5.7). The *EGFR* R222C mutation had a frequency of 1.5% in the TCGA cohort (95% CI: 0.3–2.8) but was not found in our cohort. As mentioned above, this may be due to the small sample size in our study compared to TCGA (n = 60 vs n = 391).

Other observations included were R175H, R248Q and R337C mutations being the most common missense *TP53* mutations in the LEB-GBM cohort, each comprising 3.3% (95% CI: -1.2–7.9; n = 2/60) of the samples (Table 1, S3 and S4 Tables). The most common *EGFR* mutations in our study were G598V (5.0%, 95% CI: -0.5–10.5; n = 3/60), A289T (3.3%, 95% CI: -1.2–7.9; n = 2/60) and R108K (3.3%, 95% CI: -1.2–7.9; n = 2/60) missense mutations (Table 1, S3 and S4 Tables). There was no significant association between concurrent *TP53* and *EGFR* mutations (21.4%; n = 3/14; p = 0.537). The most common *PIK3CA* mutation was the H1047R missense mutation (3.3%, 95% CI: -1.2–7.9; n = 2/60). The most common *SCN9A* mutations were E1974K (6.7%, 95% CI: 0.4–13.0; n = 4/60), S1972R (5.0%, 95% CI: -0.5–10.5; n = 3/60) and S1975K (3.3%, 95% CI: -1.2–7.9; n = 2/60) missense mutations (S3 and S4 Tables). There was no significant association between concurrent *SCN9A* and *IDH1* mutations (p = 0.999). There was also no significant association between concurrent *IDH1* and *TP53* mutations (p = 0.065).

We then explored the associations between the presence of a recurrent mutation and demographic or clinical characteristics of patients including tumor localization, age at diagnosis and recurrence. We found that the *NLRP5* gene was the only gene associated with decreased overall survival among the LEB-GBM cohort (p = 0.005). Increase in GBM size was associated with mutation in PIK3CA genes (5.3 cm vs 4.3 cm; p = 0.01). We also noted that 68.8% mutations in PIK3CA were associated with response to adjuvant TMZ treatment (p = 0.02; n = 11/16). We also found that *PTEN* mutations were significantly associated with having received concurrent TMZ (p = 0.003; n = 13/20).

We also checked for association between GBM location and gene mutations. Indeed, there was a significant association between *EGFR* mutations and GBM location in the temporal lobe (p = 0.008; n = 1/17). Also, samples with mutations in *NF1* gene were significantly associated with tumors in the temporal lobe of the brain (p = 0.02; n = 15/26). *SCN9A* mutations were significantly associated with tumors in the occipital lobe (p = 0.02; n = 6/21) and age (mean age of 38 years for mutated samples compared to 52 years for non-mutated samples, p<0.012).

Other significant observations were the following: *PCDH11X* mutations and tumors in other locations (ie, corpus callosum or basal ganglia) (p = 0.007; n = 3/20); *Kel* mutations and tumors in the temporal lobe (p = 0.04; n = 3/3); *IDH2* mutations and male gender (p = 0.02; n = 4/4), tumors in the frontal lobe (p = 0.01; n = 4/4), parietal lobe (p = 0.006; n = 4/4), and multifocality (p = 0.005; n = 4/4); *ZNF99* mutations and tumors in the frontal lobe (p = 0.04; n = 4/5). However, because the number of events was low (with an arbitrary cut-off of less than 5 events), we considered these associations non-significant.

Remaining demographic and clinical characteristics did not show significance with any of the recurrent mutations in our samples from the LEB-GBM cohort.

## Discussion

In this study, we identified 360 somatic alterations in GBM-associated genes based on WES data from 60 Lebanese patients with GBM (LEB-GBM). In our study, mutations in the *ATRX* gene were found in more than two-thirds of the LEB-GBM samples (70.0%, 95% CI: 58.4–81.6; n = 42/60). The prognostic significance of *ATRX* has been implicated in many studies [9, 20, 21]. However, according to TCGA data, mutations in *ATRX* were only present in 10.5% of the samples (95% CI: 7.4–13.5). This discrepancy was significant and could be attributed to our different population genetic makeup.

Of noteworthy importance, we found that 31.7% of LEB-GBM patients had *PCDH11X* mutations (95% CI: 19.9–43.4; n = 19/60) (S3 and S4 Tables). While there is little evidence concerning the role of PCDH11X in GBM, other protocadherin family members, such as PCDH-γ-A11, have been associated with astrocytomas, glioblastomas and glioma cell lines [22]. *PCDH11X* mutations were not present among the TCGA samples. This difference suggests that further studies need to investigate the role of PCDH11X mutations in GBM, especially in our population.

*PTEN* mutations are also common among GBM, mainly primary GBM, and these mutations are usually concurrent with LOH 10q [23]. In the TCGA cohort, *PTEN* mutations represented 35.0% of GBM patients (95% CI: 30.3–39.8; n = 137/391) with the R233* nonsense mutation being the most common *PTEN* mutation. In our study, the most common mutations were missense mutations, most frequently the I101T mutation (3.3%, 95% CI: -1.2–7.9; n = 2/60) (S4 Table). Additionally, we found an association between those who had a *PTEN* mutation and having received concurrent TMZ (p = 0.003; n = 13/20). TMZ has been suggested to be a potentially beneficial treatment option for GBM patients with *PTEN*-null mutations, given that *PTEN* mutations affect homologous recombination events [24]. Mutations in the *PTEN* gene have also been linked to GBM progression and poor prognosis [25]. However, it is unclear whether *PTEN* mutations were associated with poor prognosis in our study.

Mutations in *TP53* tumor suppressor gene were also common in the LEB-GBM cohort (26.7%, 95% CI: 15.5–37.9; n = 16/60). In comparison to published TCGA data, *TP53* is the most frequently mutated gene in GBM, detected in 41.8% of the tumors [26]. Interestingly, TCGA data showed that *TP53* mutations occurred mainly in primary GBM given that most of the tumor samples were predominantly primary GBMs [26]. The most common missense *TP53* mutations in the LEB-GBM cohort were R175H, R248Q and R337C mutations, each comprising 3.3% (95% CI; -1.2–7.9; n = 2/60) of the samples. According to the TCGA data, R175H and R248Q mutations were present in 2.0% (95% CI: 0.6–3.4; n = 8/391) and 1.8% (95% CI: 0.5–3.1; n = 7/391) of the samples, respectively. However, no R337C mutations were detected in the TCGA cohort.

A quarter of our samples had alterations in the *NF1* gene (25.0%, 95% CI: 14.0–36.0; n = 15/60). Several studies have demonstrated that the *NF1* gene, a tumor suppressor gene, is commonly mutated in GBM [12, 27]. In the TCGA cohort, mutations in the *NF1* gene were found in 13.0% of the samples (95% CI: 9.7–16.4; n = 51/391). Based on a study analysis of TCGA data, *NF1* somatic mutations occurred in 14% of the tumors [26]. Missense mutations among tumor samples from the TCGA study represented 31.6% of *NF1* mutations [26]. Although experimental data shows that loss of *NF1* in mice results in increased cell proliferation, loss of *NF1* does not necessarily result in the formation of astrocytomas [28]. Therefore, GBMs associated with *NF1* mutations might be influenced by other genetic or environmental factors.

*EGFR* mutations are more likely associated with aggressive GBM, and are overexpressed in the small cell GBM variant [29]. The most common genetic variant described is the *EGFR* variant III (*EGFR*vIII) mutant, which is the result of an in-frame deletion of 801 base pairs in the extracellular domain coding sequence [30, 31]. *EGFR* amplification is mostly associated with *EGFR*vIII overexpression, and this association is an indicator of poor prognosis [32]. Therefore, it is necessary to understand the *EGFR* mutation status among different population groups. While our findings demonstrate that the *EGFR* gene is mutated in 23.3% of patients (95% CI: 12.6–34.0; n = 14/60), the *EGFR* gene was mutated in 27.1% of the samples from the TCGA cohort (95% CI; 22.7–31.5, n = 106/391). Most *EGFR* mutations among our cohort were missense mutations, most commonly in G598V (5.0%, 95% CI: -0.5–10.5; n = 3/60), A289T (3.3%, 95% CI: -1.2–7.9; n = 2/60) and R108K (3.3%, 95% CI: -1.2–7.9; n = 2/60). G598V mutations were also common in the TCGA cohort, representing 3.3% of the cases (95% CI: 1.5–5.1; n = 13/391). The most common missense mutation in the TCGA cohort was A289V, comprising 3.8% of patients with *EGFR* mutations cases (95% CI: 1.9–5.7; n = 15/391). In our study, *EGFR* A289V mutations represented 1.7% of LEB-GBM samples (95% CI: -1.6–4.9). In our study, 21.4% of patients with *EGFR* mutations also had *TP53* mutations. However, the relationship between EGFR and TP53 mutations in the LEB-GBM cohort was non-significant. Nevertheless, it is important to denote this relationship, as simultaneous *EGFR* and *TP53* mutations are associated with worse prognosis for patients with primary GBM [33].

The PI3 kinase complex, consisting of the catalytic component (PIK3CA) and the regulatory component (PIK3R1), has also been implicated in many cancers [34, 35]. *PIK3CA* mutations are also significantly associated with GBM [36], especially in cell proliferation, migration and invasion [36, 37]. We sought to investigate the prevalence of *PIK3CA* mutations in Lebanese GBM patients and found that there were 16 *PIK3CA* mutations distributed among 13 patients, accounting for 21.7% of all patients (95% CI: 11.2–32.1). The majority of these *PIK3CA* mutations were missense mutations, with the most common missense mutation being H1047R. In comparison to the TCGA cohort, approximately 10.2% of the cases tested had a mutation in the *PIK3CA* gene (95% CI: 7.2–13.2; n = 40/391), with the most common mutations being missense mutations E545K and R88Q found in 3 patients each. On the other hand, *PIK3R1* mutations were found in 13.3% of the LEB-GBM patients (95% CI: 4.7–21.9) compared to 11.0% of the patients in the TCGA cohort (95% CI; 7.9–14.1). The most common mutation in the TCGA cohort was the G376R missense mutation present in 1.3% of patients with *PIK3R1* mutations (95% CI: 0.2–2.4; n = 5/391), but this mutation was not seen among our LEB-GBM cohort.

There has also been increasing evidence on the association of ion channel mutations and prognosis among GBM patients. More specifically, sodium ion channel mutations are significantly associated with decreased survival [38]. *SCN9A* mutations are not present in the TCGA cohort. However, *SCN9A* mutations were common in our study, with 21.7% of LEB-GBM samples having these mutations (95% CI: 11.2–32.1; n = 13/60). The most common mutations were missense mutations, namely E1974K, S1972R and S1975K mutants. In a study by Joshi et al. (2011), the mean age for patients with ion channel mutations was 49.4 years [38]. The authors also found that samples with *IDH1* mutations were not associated with sodium channel mutations [38]. Consistent with this study by Joshi et al. (2011), only one patient in our study with an IDH1 mutation also had a *SCN9A* mutation, rendering this relationship non-significant [38].

Isocitrate dehydrogenase 1 and isocitrate dehydrogenase 2 are encoded by the genes *IDH1* and *IDH2*, respectively. Mutations in the *IDH1* and *IDH2* genes usually occur in low-grade gliomas as well as secondary GBMs [39–41]. GBM patients with *IDH1* and *IDH2* mutations have a better prognosis compared to those with wild-type IDH [40]. IDH mutations were prevalent among the LEB-GBM cohort, with the most common mutation being the R132H mutation. While *IDH1* mutations were present in 6.6% of the samples in the TCGA cohort (95% CI: 4.2–9.1), R132H was still the most common missense mutation among this sample subset, representing 88.5% of all patients with *IDH1* mutations (n = 23/26). This mutation at codon 132 is shown to be a strong prognostic indicator among patients with Grade II to Grade IV gliomas [42, 43]. Interestingly, it has also been demonstrated that *IDH1* mutations are highly associated with *TP53* mutations, suggesting that *IDH1* mutations may be associated with early events in GBM tumorigenesis [44]. In our LEB-GBM cohort, *TP53* mutations were found among 4 patients with *IDH1* mutations, but this association was non-significant. This association also deemed non-significant in a study by Balss et al. (2008) where the authors used a direct sequencing method in 134 GBM samples from adult and pediatric population groups [43].

Another pathway involving PDGF and its receptor have also been implicated in normal glial cell development [45]. Any dysregulation in this pathway can contribute to tumorigenesis. GBMs, especially primary GBMs, regularly exhibit an autocrine loop in the PDGF/PDGFR signaling axis that is not present in normal brain tissues [46, 47]. More specifically, mutations in the *PDGFRA* gene are seen in all GBM subtypes but are most apparent in the proneural subtype [27]. In the TCGA cohort, *PDGFRA* mutations were present in 6.4% of samples (95% CI: 4.0–8.8). In our analysis, *PDGFRA* mutations were present in 15.0% of LEB-GBM patients (95% CI: 6.0–24.0). Missense mutations were most commonly found in our cohort. The most common missense mutations in the TCGA cohort were L655F and E229K, each of which was present in 1.7% of patients.

Studies have also shown that glioblastomas and other types of gliomas tend to have low frequencies of *BRAF* mutations [48, 49]. A rare GBM variant, epithelioid GBM, however, harbors high frequencies of *BRAF* V600E mutations, particularly in the pediatric and young adult subsets [50]. In our analysis, around 10.0% of the Lebanese GBM patients had *BRAF* mutations (95% CI: 2.4–17.6), with missense mutations of the V600E type accounting for 3.3% of the samples (95% CI: -1.2–7.9; n = 2/60). While glioblastomas tend to have a low frequency of *BRAF* mutations [48], BRAF inhibitors might potentially be useful in this population subset [51].

This study has certain limitations. While this study was an explorative study, we did not compare the genetic landscape in Lebanese patients with GBM and those without GBM. The lack of a reference genome was therefore a major limitation in our study. While our sample size was large enough for comparative analysis with existing cohorts, it was not large enough for further subgroup analyses. The comparison with non-GBM patients and other cohorts could be further explored in future studies with larger sample sizes and a wider array of GBM-related genes.

Ultimately, this is the first report to demonstrate variant detection in Lebanese patients with GBM. Taken together, this report confirms several genetic associations with GBM and highlights the need for further studies with larger sample sizes to elucidate the mechanisms of proliferation, invasion and treatment resistance in the context of GBM. The mechanisms, manifested as dysregulations in signaling pathways, may potentially be used as targets for treatment of GBM. Because most of these mutations are found simultaneously given the heterogeneity in GBM samples, multi-drug regimens would be necessary to kill GBM cells. Several barriers to treatment, such as the impermeability of the BBB and the different mechanisms of drug resistance, should be addressed as well. It is therefore very critical to understand the pathophysiology of GBM and conduct thorough genomic analyses to molecularly characterize GBM and find the optimal drug regimen for use in clinical practice.

## Supporting information

S1 Table Clinical characteristics of 60 Lebanese patients with glioblastoma. (DOCX)

S2 Table List of mutations per sample. Note that “Nonstop_Mutation” and “Translation_Start_Site” were not included in downstream analysis.
(XLSX)

S3 Table List of mutations among LEB-GBM cohort. (XLSX)

S4 Table Comparison of all GBM gene mutations between the LEB-GBM cohort and the TCGA cohort. (DOCX)
