# Supplementary material for: Correlation of genetic alterations by whole-exome sequencing with clinical outcomes of glioblastoma patients from the Lebanese population
Source: PLoS One. 2020 Nov 25;15(11):e0242793. doi: 10.1371/journal.pone.0242793 (PMC7688136; doi:10.1371/journal.pone.0242793)
Supplement: S1 Table — (DOCX) [file pone.0242793.s001.docx]

| **S1 Table. Clinical characteristics of 60 Lebanese patients with glioblastoma.** | |
| --- | --- |
| Characteristic | Number of patients (%)* |
| *Sex* |  |
| Male | 37 (61.7) |
| Female | 23 (38.3) |
| *Age in years (mean ± SD)* | 51.0 ± 17.2 |
| *Headache* |  |
| Yes | 10 (16.7) |
| No | 50 (83.3) |
| *Loss of consciousness* |  |
| Yes | 5 (8.3) |
| No | 55 (91.7) |
| *Motor deficit(s)* |  |
| Yes | 21 (35.0) |
| No | 39 (65.0) |
| *Sensory deficit(s)* |  |
| Yes | 4 (6.7) |
| No | 56 (93.3) |
| *Cranial nerve deficit(s)* |  |
| Yes | 3 (5.0) |
| No | 57 (95.0) |
| *Speech difficulties* |  |
| Yes | 6 (10.0) |
| No | 54 (90.0) |
| *Epilepsy* |  |
| Yes | 8 (15.3) |
| No | 52 (86.7) |
| *Use of anti-epileptic drug(s)* |  |
| Yes | 54 (91.5) |
| No | 5 (8.5) |
| *Localization* |  |
| Frontal | 24 (40.0) |
| Parietal | 16 (26.7) |
| Temporal | 16 (26.7) |
| Occipital | 6 (10.0) |
| Other^b^ | 4 (6.7) |
| *Size of tumor in centimeters (mean ± SD)* | 4.6 ± 1.4 |
| *Surgical resection* |  |
| Yes | 21 (35.6) |
| No | 38 (64.4) |
| *Radiation* |  |
| Yes | 60 (100.0) |
| No | 0 (0.0) |
| *Concurrent TMZ* |  |
| Yes | 52 (88.1) |
| No | 7 (11.9) |
| *Adjuvant TMZ* |  |
| Yes | 46 (92.0) |
| No | 4 (8.0) |
| *Tumor recurrence* |  |
| Yes | 55 (94.8) |
| No | 3 (5.2) |
| *Time to recurrence in months (mean ± SD)* | 10.6 ± 22.4 |
| *Follow-up duration in months (mean ± SD)* | 19.8 ± 23.7 |
| *Overall survival in months (mean ± SD)* | 17.3 ± 16.2 |

*Percentage values are reflective of the number of patients with corresponding characteristics out of the total cohort of those with reported values

^b^Includes tumors in the basal ganglia and/or corpus callosum

TMZ: temozolomide
