# Supplementary material for: Correlation of genetic alterations by whole-exome sequencing with clinical outcomes of glioblastoma patients from the Lebanese population
Source: PLoS One. 2020 Nov 25;15(11):e0242793. doi: 10.1371/journal.pone.0242793 (PMC7688136; doi:10.1371/journal.pone.0242793)
Supplement: S4 Table — (DOCX) [file pone.0242793.s004.docx]

| **S4 Table. Comparison of all GBM gene mutations between the LEB-GBM cohort and the TCGA cohort.** | | | | | | | |
| --- | --- | --- | --- | --- | --- | --- | --- |
| Gene | Mutation | Type of mutation | Number of affected samples (n=60) | % affected samples (n=60)^a^ | 95% CI (LEB-GBM cohort)^a^ | TCGA % (n=391)^b^ | 95% CI (TCGA cohort)^b^ |
| ***ATRX*** | All | − | **42** | **70.0** | **58.4-81.6** | **10.5** | **7.4-13.5** |
|  | E929Q | Missense | 38 | 63.3 | 51.1-75.5 | 0.0 | 0.0-0.0 |
| ***PCDH11X*** | All | − | **19** | **31.7** | **19.9-43.4** | **0.0** | **0.0-0.0** |
|  | *S958L* | Missense | 14 | 23.3 | 12.6-34.0 | 0.0 | 0.0-0.0 |
| ***PTEN*** | All | − | **18** | **30.0** | **18.4-41.6** | **35.0** | **30.3-39.8** |
|  | *I101T* | Missense | 2 | 3.3 | -1.2-7.9 | 0.3 | -0.2-0.8 |
| ***TP53*** | All | − | **16** | **26.7** | **15.5-37.9** | **31.7** | **27.1-36.3** |
|  | *R175H* | Missense | 2 | 3.3 | -1.2-7.9 | 2.0 | 0.6-3.4 |
|  | *R248Q* | Missense | 2 | 3.3 | -1.2-7.9 | 1.8 | 0.5-3.1 |
|  | *R337C* | Missense | 2 | 3.3 | -1.2-7.9 | 0.0 | 0.0-0.0 |
|  | R248W | Missense | 1 | 1.7 | -1.6-4.9 | 1.3 | 0.2-2.4 |
|  | R282W | Missense | 0 | 0.0 | 0.0-0.0 | 1.3 | 0.2-2.4 |
| ***NF1*** | All | − | **15** | **25.0** | **14.0-36.0** | **13.0** | **9.7-16.4** |
| ***EGFR*** | All | − | **14** | **23.3** | **12.6-34.0** | **27.1** | **22.7-31.5** |
|  | *G598V* | Missense | 3 | 5.0 | -0.5-10.5 | 3.3 | 1.5-5.1 |
|  | *A289T* | Missense | 2 | 3.3 | -1.2-7.9 | 1.0 | 0.0-2.0 |
|  | *R108K* | Missense | 2 | 3.3 | -1.2-7.9 | 1.0 | 0.0-2.0 |
|  | A289V | Missense | 1 | 1.7 | -1.6-4.9 | 3.8 | 1.9-5.7 |
|  | R222C | Missense | 0 | 0.0 | 0.0-0.0 | 1.5 | 0.3-2.8 |
|  | A289D | Missense | 0 | 0.0 | 0.0-0.0 | 1.3 | 0.2-2.4 |
| ***PIK3CA*** | All | − | **13** | **21.7** | **11.2-32.1** | **10.2** | **7.2-13.2** |
|  | *H1047R* | Missense | 2 | 3.3 | -1.2-7.9 | 0.5 | -0.2-1.2 |
|  | E545K | Missense | 0 | 0.0 | 0.0-0.0 | 0.8 | -0.1-1.6 |
|  | R88Q | Missense | 0 | 0.0 | 0.0-0.0 | 0.8 | -0.1-1.6 |
| ***SCN9A*** | All | − | **13** | **21.7** | **11.2-32.1** | **0.0** | **0.0-0.0** |
|  | *E1974K* | Missense | 4 | 6.7 | 0.4-13.0 | 0.0 | 0.0-0.0 |
|  | *S1972R* | Missense | 3 | 5.0 | -0.5-10.5 | 0.0 | 0.0-0.0 |
|  | *S1975K* | Missense | 2 | 3.3 | -1.2-7.9 | 0.0 | 0.0-0.0 |
| ***IDH1*** | All | − | **9** | **15.0** | **6.0-24.0** | **6.6** | **4.2-9.1** |
|  | *R132H* | Missense | 6 | 10.0 | 2.4-17.6 | 5.9 | 3.6-8.2 |
| ***PDGFRA*** | All | − | **9** | **15.0** | **6.0-24.0** | **6.4** | **4.0-8.8** |
| ***PIK3R1*** | All | − | **8** | **13.3** | **4.7-21.9** | **11.0** | **7.9-14.1** |
|  | G376R | Missense | 0 | 0.0 | 0.0-0.0 | 1.3 | 0.2-2.4 |
| ***LZTR1*** | All | − | **7** | **11.7** | **3.5-19.8** | **4.6** | **2.5-6.7** |
| ***NLRP5*** | All | − | **7** | **11.7** | **3.5-19.8** | **0.0** | **0.0-0.0** |
|  | *N255S* | Missense | 2 | 3.3 | -1.2-7.9 | 0.0 | 0.0-0.0 |
| ***BRAF*** | All | − | **6** | **10.0** | **2.4-17.6** | **2.6** | **1.0-4.1** |
|  | *V600E* | Missense | 2 | 3.3 | -1.2-7.9 | 1.3 | 0.2-2.4 |
| ***COL1A2*** | All | − | **6** | **10.0** | **2.4-17.6** | **0.0** | **0.0-0.0** |
| ***STAG2*** | All | − | **6** | **10.0** | **2.4-17.6** | **4.3** | **2.3-6.4** |
| ***TERT*** | All | − | **4** | **6.7** | **0.4-13.0** | **2.8** | **1.2-4.5** |
|  | *R268C* | Missense | 1 | 1.7 | -1.6-4.9 | 0.0 | 0.0-0.0 |
|  | *R622C* | Missense | 1 | 1.7 | -1.6-4.9 | 0.0 | 0.0-0.0 |
|  | *R743W* | Missense | 1 | 1.7 | -1.6-4.9 | 0.0 | 0.0-0.0 |
| ***H3F3A*** | All | − | **3** | **6.7** | **-0.5-10.5** | **0.8** | **-0.1-1.6** |
|  | K28M | Missense | 2 | 3.3 | -1.2-7.9 | 0.0 | 0.0-0.0 |
| ***HIST1H3B*** | All | − | **2** | **3.3** | **-1.2-7.9** | **0.0** | **0.0-0.0** |
| ***IDH2*** | All | − | **1** | **1.7** | **-1.6-4.9** | **0.5** | **-0.2-1.2** |
| ^a^The denominator used for the percentages values in these columns was 60, reflective of the number of samples tested  ^b^The denominator used for the percentages values in these columns was 391, reflective of the number of cases tested for gene mutations | | | | | | | |
